# Studying naturalistic human communication using dual-EEG and audio-visual recordings

**DOI:** 10.1016/j.xpro.2023.102370

**Published:** 2023-07-07

**Authors:** Sara Mazzini, Judith Holler, Linda Drijvers

**Affiliations:** 1 The Communicative Brain, Neurobiology of Language Department, Max Planck Institute for Psycholinguistics, Nijmegen 6525XD, the Netherlands; 2 Communication in Social Interaction, Max Planck Institute for Psycholinguistics, Nijmegen 6525XD, the Netherlands; 3 Communication in Social Interaction, Donders Centre for Cognition, Donders Institute for Brain, Cognition and Behavior, Radboud University, Nijmegen 6525GD, the Netherlands; 4 The Communicative Brain, Donders Centre for Cognition, Donders Institute for Brain, Cognition and Behavior, Radboud University, Nijmegen 6525GD, the Netherlands

**Keywords:** Neuroscience, Cognitive Neuroscience

## Abstract

We present a protocol to study naturalistic human communication using dual-electroencephalography (EEG) and audio-visual recordings. We describe preparatory steps for data collection including setup preparation, experiment design, and piloting. We then describe the data collection process in detail which consists of participant recruitment, experiment room preparation, and data collection. We also outline the kinds of research questions that can be addressed with the current protocol, including several analysis possibilities, from conversational to advanced time-frequency analyses.

For complete details on the use and execution of this protocol, please refer to Drijvers and Holler (2022).^1^

## Highlights

• A protocol to collect dual-EEG and audio-visual data to study human communication
• Suitable to address a wide variety of research questions and analysis goals
• Detailed instructions on how to combine dual-EEG and audio-visual recording equipment
• Dual-EEG-specific procedures: experiment design, data collection, and analysis

## Abstract (editor highlights)

Publisher’s note: Undertaking any experimental protocol requires adherence to local institutional guidelines for laboratory safety and ethics.

## Graphical abstract

**Figure:**
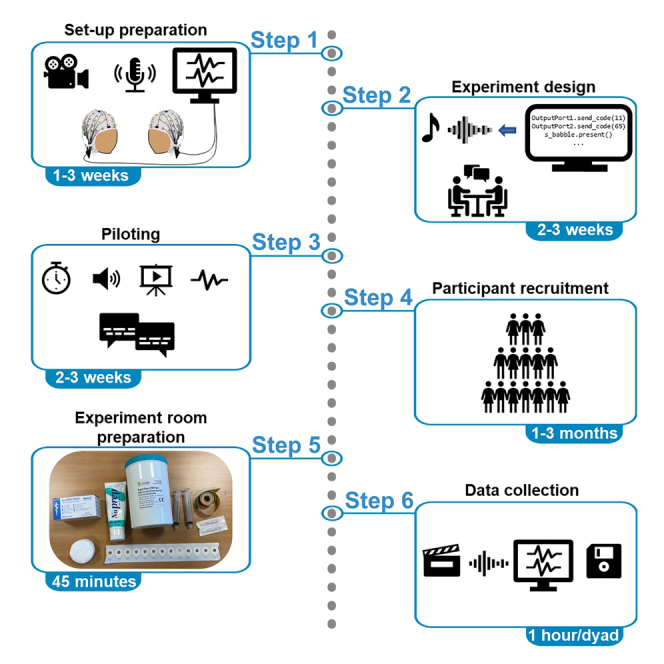

## Teaser

We present a protocol to study naturalistic human communication using dual-electroencephalogram and audio-visual recordings. We describe preparatory steps for data collection including setup preparation, experiment design, and piloting. We then describe the data collection process in detail which consists of participant recruitment, experiment room preparation, and data collection. We also outline the kinds of research questions that can be addressed with the current protocol, including several analysis possibilities, from conversational to advanced time-frequency analyses.

## Before you begin

In the last two decades, a multitude of studies in the field of cognitive neuroscience started to investigate social interaction and the cognitive processes behind it.^1^^,^^2^ Up until then, most research was limited to the study of the brain of a single participant involved in social interaction, therefore not capturing the dyadic or group aspect of social interaction.^3^ The advent of hyperscanning techniques, such as dual-electroencephalography (EEG), offered the possibility to measure the brain activity of multiple individuals at the same time, enabling the investigation of the neural dynamics beyond a single participant and between interacting brains.^4^^,^^5^^,^^6^^,^^7^^,^^8^ Due to its high temporal resolution, collecting dual-EEG in combination with audio-visual data also offers the opportunity to directly relate the behavioral and neural aspects of social interaction to each other.

This protocol describes the necessary steps for collecting dual-EEG and audio-visual data to study human face-to-face communication, as well as its underlying intra- and inter-brain neural dynamics. The collected data will be suitable for different types of analyses, such as conversation and gesture analyses, intra-brain spectral and time-frequency analyses, as well as intra- and inter-brain connectivity and coherence analyses. More specifically, we describe the setup and procedure implemented in two experiments, to illustrate how the protocol can be expanded to address multiple research questions on human communication.

**Figure 1:**
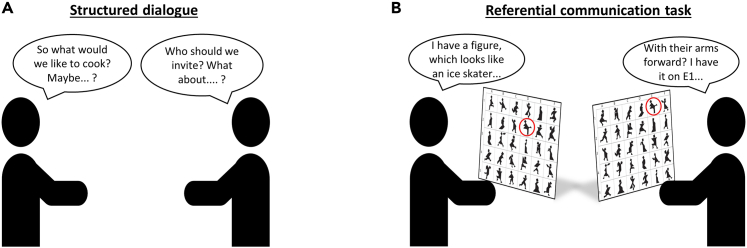
Tasks implemented and described in the protocol (A) Structured dialogue to plan an event: a dinner, a holiday or a festival.^9^ (B) Referential communication task. The geometrical figures were printed and attached to a grid in front of each participant: the only difference between the figures of the two participants was their position on the grid. The goal of the task was to move each figure to a shared position, so that by the end of the task, participants’ grids would look exactly the same (see Clark & Wilkes-Gibbs^34^ and Holler & Wilkin^35^ for task details).

We first refer to the study by Drijvers & Holler,^9^ one of the first dual-EEG studies of multimodal face-to-face interaction. The authors investigated the influence of spatial orientation and the visibility of bodily signals on intra- and inter-brain neural synchrony during dyadic interactions. In this work, participants engaged in a structured conversation, during which they alternated between the roles of speaker and listener while planning an event (Figure 1A). An auditory cue was presented to participants every 60 s to indicate the start of the following conversational turn. Participants communicated under three different conditions: face-to-face, back-to-back, and face-to-face with a visual occluder.

Secondly, we describe how the protocol has been expanded to study task-oriented dialogue and to directly relate speech annotations to the recorded neural activity (Mazzini, Holler, Hagoort & Drijvers, *in preparation*). In this case, participants performed a referential communication task: they engaged in a conversation about a set of geometrical figures (Figure 1B), describing their (spatial) characteristics until they reached a shared conceptual understanding of each figure. An auditory cue was presented every 10 s to guide participants’ speaking turns and, in one condition, multi-talker babble was presented to simulate noisy communicative settings (i.e., a cocktail party effect^10^). In both experiments, participants' demographics as well as behavioral questionnaires were also collected, to potentially model inter-dyad differences in neural activity.

The setup and procedure specific to each study are described in separate paragraphs. An overview of the main analysis steps specific to dual-EEG data is also provided at the end of the protocol.

### Institutional permissions

This protocol is based on the study by Drijvers & Holler^9^ involving human participants. The study was conducted in accordance with the Declaration of Helsinki and approved by the Ethics Committee of the Social Sciences department of Radboud University.

Since audio-visual recordings serving multimodal analyses of social interactions are not anonymized, the data collected are classed as personally sensitive information and need to be 1) approved by a local ethics committee, 2) processed, analyzed, and stored in a way that protects the data from access by unauthorized third parties and 3) disseminated only with participants explicit, informed consent and in line with GDPR rules.

### Setup preparation

**Timing: 1–3 weeks**

1. Experiment room (Figure 2)
  a. Define the places in which participants will be seated during the experiment:
    i. Make sure the same lighting conditions apply to the selected places.
    ii. Place two chairs on the selected places.
    iii. Mark the places with tape on the floor so that the chairs can be placed in the same position every time the experiment is run.
  b. Place additional tables or counters for the EEG setup and supplies.
  c. **Structured dialogue:** Prepare a visual occluder (e.g., a panel) and decide where to place it during the face-to-face occluded condition (Figure 2B). Mark the placing with tape on the floor so that the occluder is placed in the same position every time the experiment is run.
  d. **Referential communication task:** Place a table between the two chairs and place one stimuli stand on each side of the table (Figure 2C).

**Figure 2:**
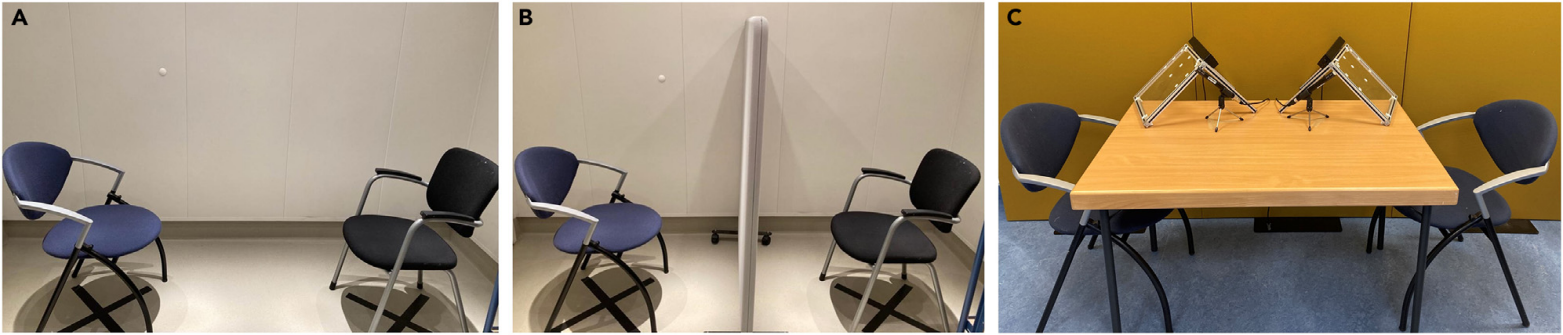
Experiment room setup (A and B) Structured dialogue setup for face-to-face and face-to-face occluded conditions, respectively. (C) Referential communication task setup, including two chairs and a table in between them, on which the microphones and the stimuli stands are positioned.
2. Dual-EEG setup (see Figure 3):
  ***Note:*** The necessary hardware, based on BrainProducts’ equipment,^11^ is listed in the key resources table. The setup includes ActiCAP snap caps with active slim electrode sets and BrainAmp DC amplifiers. We recommend using active electrodes to reduce preparation time, since they perform better at higher impendence levels^12^: impedances up to 25 kOhm are considered fine with active electrodes, while for passive electrodes the criteria is lower (< 5kOhm).^13^ For other manufacturer and/or equipment features, consult Barraza et al.^14^ and/or the Brain Products’ dedicated pages.^15^^,^^16^
  a. Connect the electrodes to the amplifiers:
    i. Connect each of the two 32-channel electrode sets, placed into two electrode caps respectively, to a ControlBox with a flat ribbon cable.
    ii. Insert the reference and the ground electrodes into their designated entries on the ControlBox. Use a separate reference and ground electrode for each participant.
    iii. Connect each control box to one amplifier and connect each amplifier to a charged PowerPack.
    iv. Connect the amplifiers to the USB2 Adapter with fiber optic cables. Connect the amplifier, and the electrode set, of the first participant (from here on referred to as participant A) to the Fiberoptic1 port of the USB2 Adapter.
    v. Connect the amplifier, and the electrode set, of the second participant (from here on referred to as participant B) to the Fiberoptic2 port of the USB2 Adapter.
      ***Note:*** Following this procedure, the EEG activity of both participants is visualized and recorded together in BrainVision Recorder, as a 64-channel recording. The first 32 electrodes visualized (electrodes 1–32) will measure the brain activity of participant A and the following 32 electrodes will measure the brain activity of participant B (electrodes 33–64). For higher-density EEG recordings (64 channels per participant), two additional 32 electrode sets can be used: each set needs to be connected to each participant ControlBox (using the *Ch.33-64 Splitter Box* entry, highlighted in yellow in Figure 3), to which an additional amplifier needs to be connected (using the *Ch. 33–64 Amplifier* entry, also highlighted in yellow in Figure 3). Each amplifier needs to be connected to a charged PowerPack and to the USB2 Adapter. In this case, the two amplifiers connected to participant A’s electrodes sets (Ch. 1–32 and Ch. 33–64) will be connected to the Fiberoptic1 and Fiberoptic2 ports, respectively; the two amplifiers connected to participant B’s electrodes sets (Ch. 1–32 and Ch. 33–64) will be connected to the Fiberoptic3 and Fiberoptic4 ports, respectively. The result of the described procedure will be a 128-channels recording, where the first 64 channels visualized (channels 1–64) will represent the brain activity of participant A and the following 64 channels (channels 65–128) will represent the brain activity of participant B.
    vi. Connect the USB2 Adapter to the recording computer with a USB cable.
    vii. Connect the USB2 Adapter to the stimulation computer with an LTP cable from the Trigger port (Figure 3).

**Figure 3:**
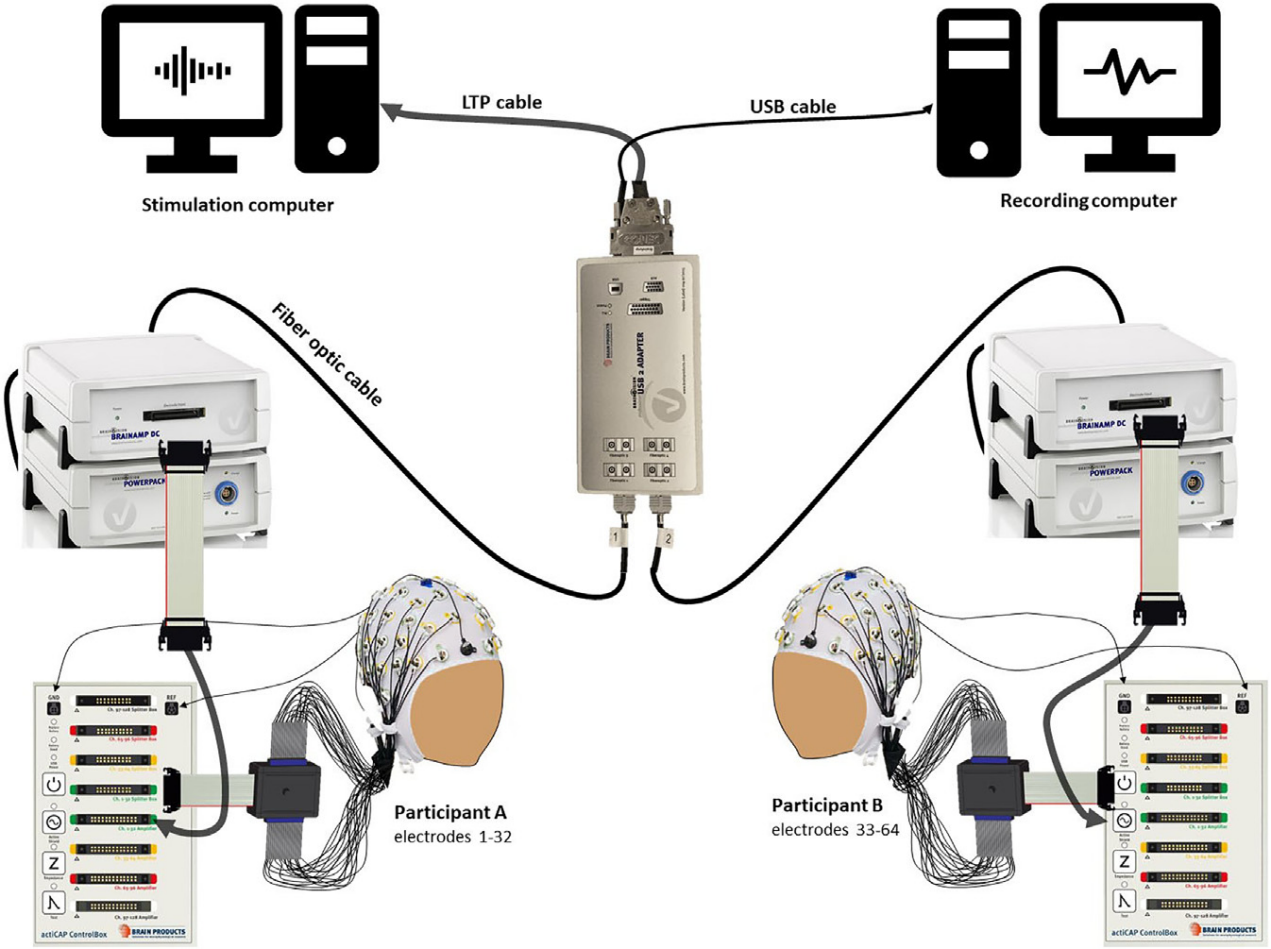
Dual-EEG setup, based on BrainProducts supplies (including active electrodes and BrainAmp DC amplifiers) Each participant’s electrode set is connected to the participant’s control box, which is connected to the participant’s amplifier. The ground and reference electrodes of each participant are inserted directly in each control box. The amplifiers need to be connected to a PowerPack battery, respectively. The amplifiers are then connected to the USB2 Adapter via optic fiber cables. The amplifier (and electrode set) connect to the Fiber Optic 1 will be read as first 32 electrodes (1–32, Participant A), whereas the amplifier (and electrode set) connected to the Fiber Optic 2 will be read as the following 32 electrodes (33–64, Participant B).
3. Recording audio-visual behavior:
  a. **Structured dialogue:** Place two cameras in front of the participants: one camera to record participant A and one camera to record participant B. If needed, the cameras can be placed on a small table (Figures 4A and 4B). Use cameras with integrated microphones (e.g., CANON HF G30).

**Figure 4:**
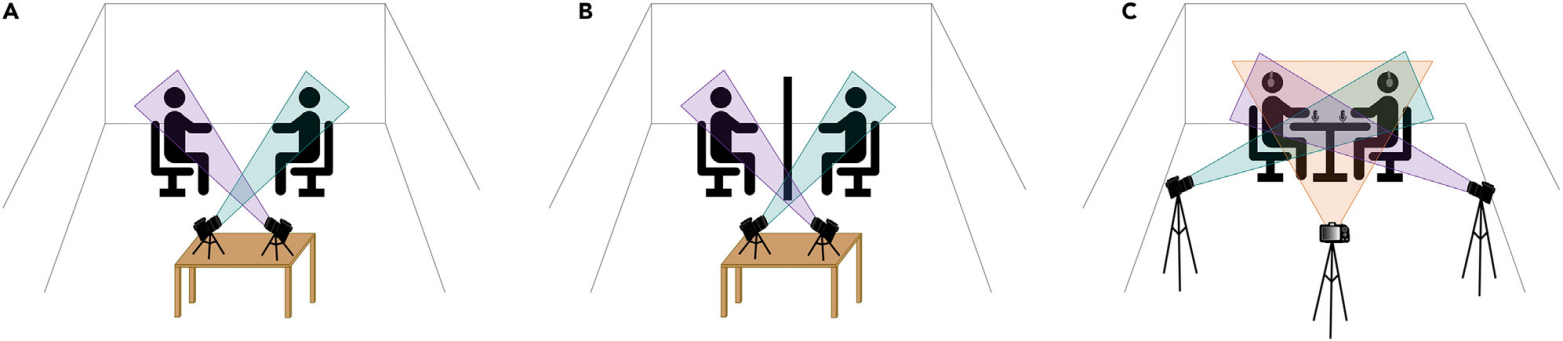
Audio-visual setup The equipment implemented to record audio-visual behavior is illustrated for both tasks. (A and B) Structured dialogue, two cameras with built-in microphones were used. Each camera captured one participant. (C) Referential communication task, three cameras and two microphones were used. Each camera captured a different view of participants: respectively, participant A, both participants and participant B.
  b. **Referential communication task:**
    i. Place three cameras around the participants’ chairs: one camera to record the front view of participant A, one camera to record the front view of participant B and one camera to record both participants from the side (see Figure 4C).
      ***Note:*** This video recording setup offers the possibility to study visual communicative signals, involving both the face and the hands. In this way, visual behavior can be studied both with an interactional point of view (e.g. behavioral alignment) and with a focus on each participant in isolation (e.g. close-ups of facial and hand gestures).
    ii. Place one microphone on the table in front of each participant. Place them on one side of the table to avoid limiting the co-participants’ visibility and each participant’s gestural space.
4. **Structured dialogue:** Auditory cue presentation.
  ***Note:*** This step is particularly relevant to study cortical tracking of speech or audio-visual behavior in relation to neural synchrony: the auditory cue and the related event marker are used to synchronize the EEG signal and the audio recording (from which the speech envelope is extracted). In this case, make sure to include one auditory cue (and its event marker) at the beginning of the EEG recording. The auditory cue can also be used throughout the task to guide the participants’ turn-taking behavior.
  a. Load the auditory cue in Presentation.
  b. Connect a speaker to the stimulation computer (or to the tone generator) for the presentation of the auditory cue.
5. **Referential communication task:** Auditory cue and multi-talker babble presentation.
  ***Note:*** You will need two audio mixers to record the auditory cues together with the participants’ conversation, but to exclude the multi-talker babble from the audio-visual recording. The auditory cues can be generated by means of an automated tone generator, which can be built with Arduino hardware products (see Figures S1 and S2 for more information).
  a. The first audio mixer serves to combine the multi-talker babble and the auditory cues, which are presented to participants via headphones.
    i. Plug the audio mixer (XENYXSO2) into a socket.
    ii. Connect the stimulation computer to the audio mixer using the LINE IN 2/3 inputs (both L and R) (Figure 5).

**Figure 5:**
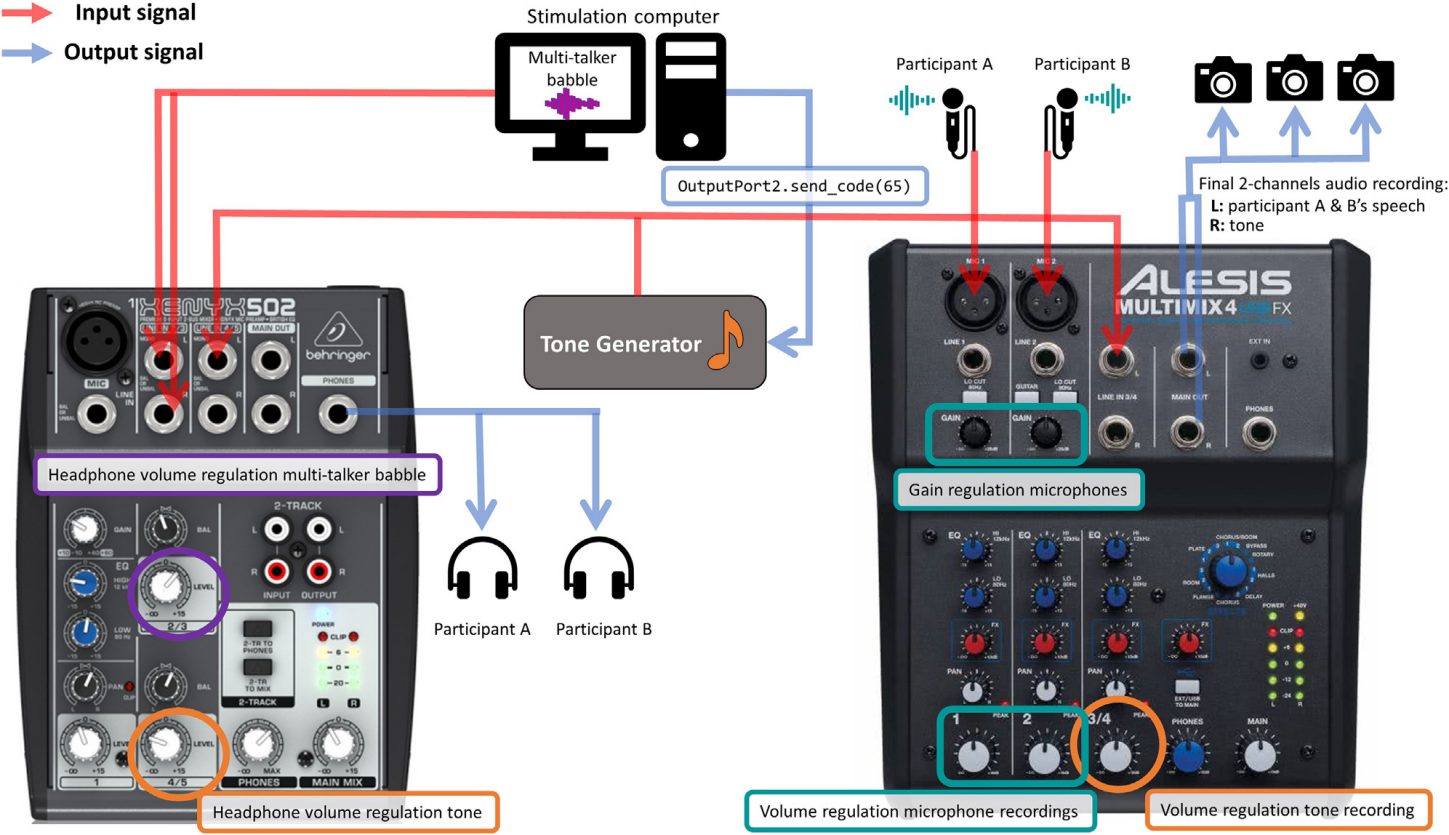
Audio mixers for the presentation of multi-talker babble The XENYXSO2 audio mixer (on the left) was implemented to combine the multi-talker babble and the tones in one signal, which was presented to the participants via headphones. The volume of the babble and of the tone can be directly regulated through the audio mixer. The ALESIS MULTIMIX4 audio mixer (on the right) was used to combine the speech signal coming from the microphones to the tones. The combined signal was then sent to each camera as microphone input.
    iii. Connect the stimulation computer to the tone generator with a USB cable and connect the tone generator to the audio mixer using the LINE 4/5 input (L) (Figure 5).
    iv. Connect the headphones with an audio-splitter cable to the audio mixer’s *phones* output.
    v. Regulate the volume of the interfering stimulus using the LEVEL 2/3 knob (Figure 5).
    vi. Regulate the volume of the auditory cue using the LEVEL 4/5 knob.
  b. The second audio mixer serves to combine the recorded speech and the auditory cue in the audio-visual recordings, excluding the interfering stimulus.
    i. Plug the audio mixer (ALESIS MULTIMIX4 USB FX) into a socket.
    ii. Connect the two microphones to the audio mixer via the inputs MIC1 and MIC2.
    iii. Connect the tone generator to the input LINE 3/4 of the audio mixer.
    iv. Connect all the cameras to the MAIN outputs of the audio mixer. The speech recording will be saved on the left channel and the auditory cue on the right channel.
    v. Select external *microphone* on the cameras’ input signal settings.
    vi. Regulate the gain of the microphones using the two GAIN knobs.
    vii. Regulate the volume of the speech recording using knobs 1 and 2.
    viii. Regulate the volume of the auditory cue using the 3/4 knob.

### Experiment design

**Timing: 2–3 weeks**

**Figure 6:**
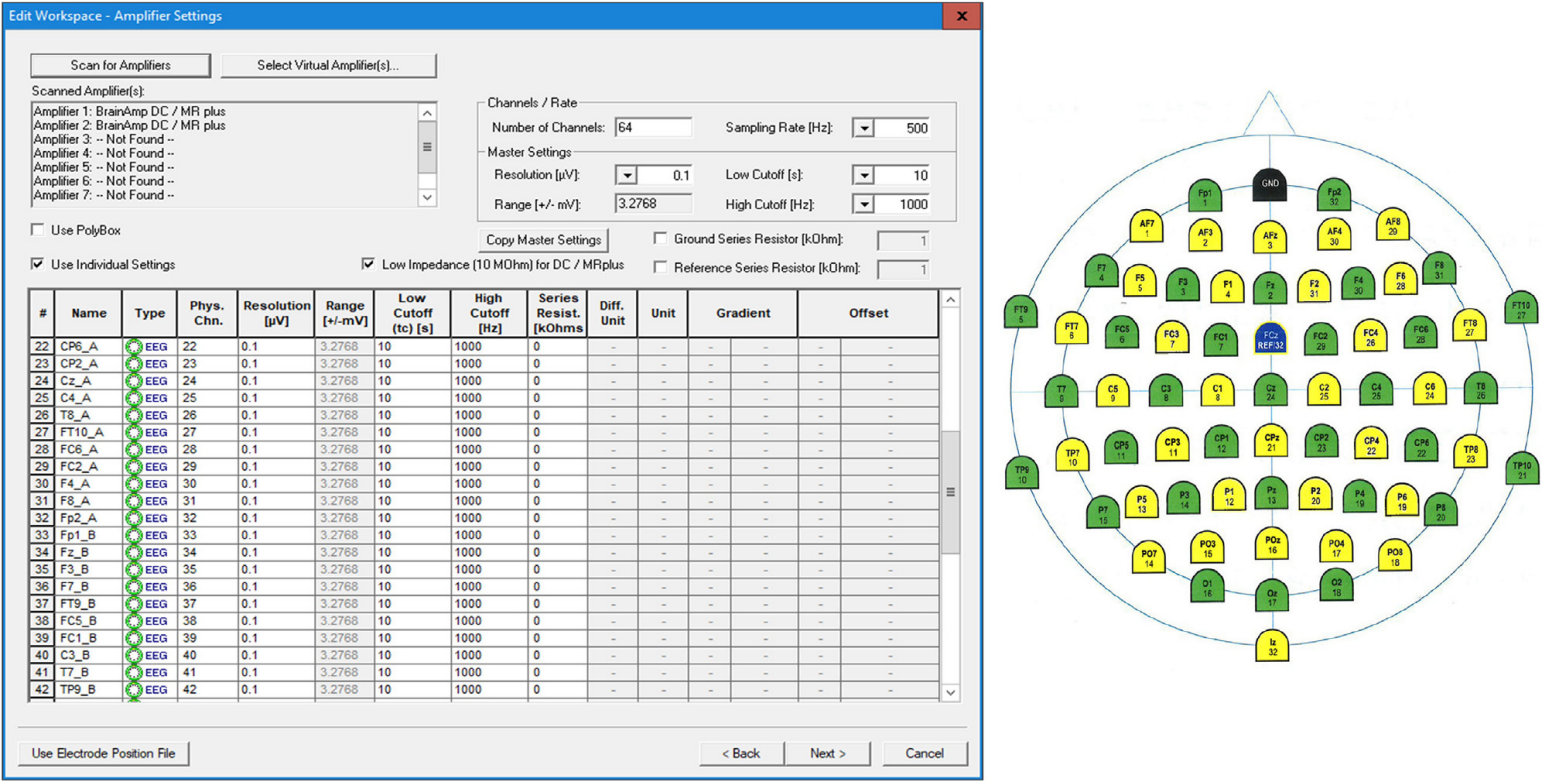
BrainVision Recorder workspace settings and ActiCAP snap cap’s electrode names and number labels Left: Channel list included in the workspace, including both electrodes from participant A and electrodes from participant B. Right: electrode cap positions of ActiCAP snap cap, for 32-channels recordings, the green electrode positions are used.
    iii. Rename the following 32 electrodes (33–64, corresponding to participant B) them as *electrodename*_B (e.g., Fz_B) (Figure 6).
    iv. Make sure that the order of the physical channels proceeds regularly from 1 to 64. If it is not the case, you can change this manually or by right-clicking on the channel table.
    v. If you use active electrodes, no channels need to be added for ground and reference electrodes, as these are plugged directly into the control box.
  i. Click on Next, and enable and define filters, if desired.
  j. Save the edited workspace.
  k. If you use passive electrodes and you check the electrode impedances on BrainVision Recorder, we suggest you to adapt the channel locations for a clearer visualization.
    i. After opening your workspace, click on the impedance button in BrainVision Recorder .
    ii. You will see that the channels of participant A and B are arranged on top of each other. For a better visualization, move the channels of participant A and B side-by-side (see Figure 7) and save the changes.

6. Select task(s) and conditions to test your research question(s):
  a. Decide if you would like to study spontaneous free conversation or if you would like to have some experimental control over participants’ conversations. For instance, by giving participants a pre-defined topic/goal (e.g., structured dialogue) or by asking them to perform a specific task (e.g., referential communication task).
    i. **Structured dialogue:** define the topics that you will ask participants to communicate about.
    ii. **Referential communication task:** define the visual stimuli you would like to use and how to present them to participants (e.g., as physical figures or objects or on a digital device such as a tablet).
  b. Determine the conditions between which the behavioral and neural activity will be compared. Select conditions which allow you to exclude potential confounding factors.
    ***Note:*** This step is particularly relevant for the investigation of inter-brain dynamics: avoid manipulating the presence of external stimuli (e.g. visual or auditory stimuli) as independent variable to exclude similar inter-brain patterns reflecting common cognitive processes elicited by the stimuli (see Hamilton^17^ for more details and problem 1 in the troubleshooting section).
  c. Determine how many blocks will be included in the experiment.
  d. Determine the roles participants will have during the experiment (e.g., one participant is the speaker and the other is the listener or alternating speaker-listener roles).
    i. For alternating speaker-listener roles, determine how participants will exchange roles/take turns (e.g., spontaneously or elicited by an auditory cue).
    ii. Determine how long the participants’ turns should be (if not spontaneous).
  e. Randomize the order of the conditions across dyads and print them in a document, to be consulted before each testing session (see Figure S3).
  f. Randomize the order of the speaker-listener roles across dyads and print them in a document, to be consulted before each testing session.
  g. Randomize the order of whether participant A or B sits in the left or right chair and print them in a document, to be consulted before each session.
7. Create auditory and visual stimuli:
  a. Select a sound to indicate the turn exchange or use an automated tone generator.
  b. Select a sound to indicate the beginning of the experiment (from here on referred to as *EEG recording cue*), this can also correspond to the first auditory turn cue.
  c. **Referential communication task**:
    i. Create your visual stimuli.
    ii. Decide how long the task should take and how many trials each condition should have. For example, in the referential communication Tangram task, each participant has 12 laminated figures (in a different order) in front of them. For a task duration between 15 and 30 min, we suggest using 30 figures per block (Mazzini, Holler, Hagoort & Drijvers, in preparation).
    iii. Print the selected stimuli on laminated paper.
    iv. Depending on your research question, the laminated stimuli can be placed on the table in front of each participant or they can be attached (with Velcro) to a 45-degree tilted stand and positioned on the side of the table in front of each participant. The second option will avoid limiting participants’ visibility of their co-participant (Figure 2C).
      ***Note:*** The stimuli could also be presented on a digital device (e.g. tablets or monitors). In this case, make sure participants will have the possibility to move the stimuli around freely.
  d. **Referential communication task:** create multi-talker babble, to simulate noisy naturalistic communication settings (e.g., cocktail party effect^10^) and to compare it to a clear speech condition.
    i. Decide how many *talkers* you would like to use.
    ii. Ask some volunteers to read out a book for 5 min and record the audio while they are reading.
    iii. Open Praat^18^ (version 6.1.42), for each talker audio: load the audio recording and trim it in short time segments (e.g., 15 s): In Praat,^18^ click *Convert > extract part > select time frame of 15 s.*
    iv. Save the extracted audio traces as .wav files. In order to obtain a multi-talker babble, in which the talkers’ speech content is different and does not follow a meaningful syntactic structure, shuffle the talkers’ audio recording in random order. Load the extracted audio traces in Praat^18^ in a randomized order and combine them into one audio trace: To achieve this, you need to select the traces in Praat,^18^ and click *Combine > Concatenate > Save as .wav file.*
    v. Repeat the same procedure for each talker recording.
    vi. To create the multi-talker babble, load the talkers’ shuffled audio traces and select them by clicking *Combine > Combine to stereo.*
    vii. Select the obtained combined audio trace, by clicking *Convert > Convert to mono.*
    viii. Save the babble as .wav file.
      ***Note:*** Keep in mind that if the target speaker and the babble speaker are of different sex, it might be easier to segregate the two audio sources, since these have different auditory characteristics, e.g. differences in pitch and intonation.^19^ As a result, it may be easier for the participant to disentangle them.

8. Program the experiment in Presentation® software (version 22.1, Neurobehavioral Systems, Inc. Berkeley, CA).
  a. Download the Presentation® software and buy a license. For more information on the different license types, consult the Neurobehavioral Systems’ dedicated page.^20^
  b. Create a scenario file using the Scenario Definition Language (see key resources table for example scripts).
    i. The scenario file describes the objects which will be used during the experiment, such as a picture or a sound:
    picture {
    text { caption = “End of the block: press Space to continue”; font_size = 40;} t_BlockEnd; x = 0; y =0;
    } p_BlockEnd;
    sound {
    wavefile { filename = “fembabble_mono.wav”; preload = true;} w_FemBabble;
    } s_FemBabble;
  c. Create three PCL-files using Program Control Language. The PCL-files serve to control the stimuli defined in the scenario file, for instance when and how the stimuli will be presented.
    i. Create one PCL file (*Subs*) containing all subroutines. A subroutine contains a determined sequence of operations, which can be accessed in your PCL code multiple times throughout the experiment. Create a subroutine for each block/condition of the experiment.
    ii. Create one PCL file (*Main*) containing the structure of the experiment, such as the calls to the subroutines/blocks of the experiment.
    iii. Create one PCL file (*Vars*) containing the variables declarations.
  d. Connect the PCL files together with the scenario file.
    i. In the scenario file’s header, add the line:
      pcl_file = “PCL_MAIN.pcl”.
    ii. In the Main PCL file add the lines:
    include “PCL_VARS.pcl”;
    include “PCL_SUBS.pcl”;
  e. Create a new experiment:
    i. Under the *Scenarios* tab, add the path to the Stimulus Directory and select the Scenario file and all other relevant files (experiment file, PCL files).
    ii. Define the active buttons in the *Settings* tab under the *Response* section. Add Space bar as an active button. This button is used to stop the current block and proceed to the following.
      ***Note:*** Active buttons are buttons which will be used during the experiment (e.g., to respond to a predetermined stimulus or to proceed to the next block) and precise information about these button presses can be obtained (e.g., when they were pressed or how many times).
    iii. In the Settings tab under the *Audio* section, you can specify more details on how to play the audio files.
      **CRITICAL:** Check the *‘Set Device and Application Volume’* option and select the desired device volume. This setting ensures that the volume of the audio does not change, even if the computer volume level changes.
    iv. In the Settings tab under the *Port* section, add one LTP output port connecting the stimulation computer to the recording computer.
    v. **Referential communication task:** add another output port (COM3) connecting the stimulation computer to the tone generator device.
    vi. Save the experiment file.
  f. Send data markers to BrainVision Recorder.
    i. Create a handle to send a marker in the Vars file:
      output_port OutputPort1 = output_port_manager.get_port(1)
    ii. Define specific marker codes for the task *‘events’* you would like to synchronize your EEG analysis to (e.g., *66* start of the task, *77* turn-exchange, *67* end of the task).
    iii. Use the line below to send a marker code when the EEG recording cue is presented, for the turn-taking and at the end of the experiment (e.g., after the space bar button press).
      OutputPort1.send_code(77);
    iv. Do the same for any other events you would like to mark. Change the number between brackets to your defined event marker codes.
  g. **Referential communication task:** tone generator
    i. Create a handle to send a command to generate the tone in the Vars file:
      OutputPort2 = output_port_manager.get_port(2)
    ii. Use the line below to send the command to generate the tone. Change the number between brackets, based on your tone generator settings:
    OutputPort2.send_code(65)

9. Create a workspace in BrainVision Recorder (version 1.23.0001, Brain Products GmbH, Gilching, Germany)
  a. Click on the *File* tab and select *New Workspace.*
  b. In the pop-up window, edit the data file settings:
    i. Select the directory where the EEG data will be saved (*Raw File Folder*)
    ii. If desired, enable the automatic filename generation and fill in the details.
  c. Click on *Next* and edit the *Amplifier Settings.*
    **CRITICAL:** In order to edit the *Amplifier Settings*, you need to have two amplifiers connected to the recording computer and both need to be turned on (and therefore connected to power packs). When you click on Scan for Amplifiers, you should see them appearing in the list as *Amplifier 1* and *Amplifier 2*).
  d. Select 64 as the number of channels and the desired sampling rate (e.g., 500 Hz).
  e. In the master settings, you can use the default settings of BrainVision Recorder: 0.1 as resolution, 10s as Low cutoff and 1000 Hz as High Cutoff.
  f. Check the options *Use Individual Settings* and *Low Impedance (10 MOhm) for DC/MRplus*, if you are using DC Amplifiers.
    ***Note:*** Electrode name and number labels and their position are assigned in the workspace. The cap manufacturer normally provides an electrode position file (BVEF file), this can be imported in BrainVision Recorder to retrieve the electrodes information. For active electrodes, the impedances can be checked directly on the electrode (i.e. red light = high impedances, green light = good impedances) and the electrode position is not necessary.
  g. Import the electrode position file (.BVEF):
    i. Click on *Use electrode position file* and select the BVEF file.
    ii. Click on *Import amplifier channel table > OK.*
    iii. Check that the channel table includes 32 electrodes, with their name and number labels.
  h. Edit the channel table:
    i. Rename the first 32 electrodes (1–32, corresponding to participant A) as
    ii. as electrodename_A (e.g., Fz_A) (Figure 6).

**Figure 7:**
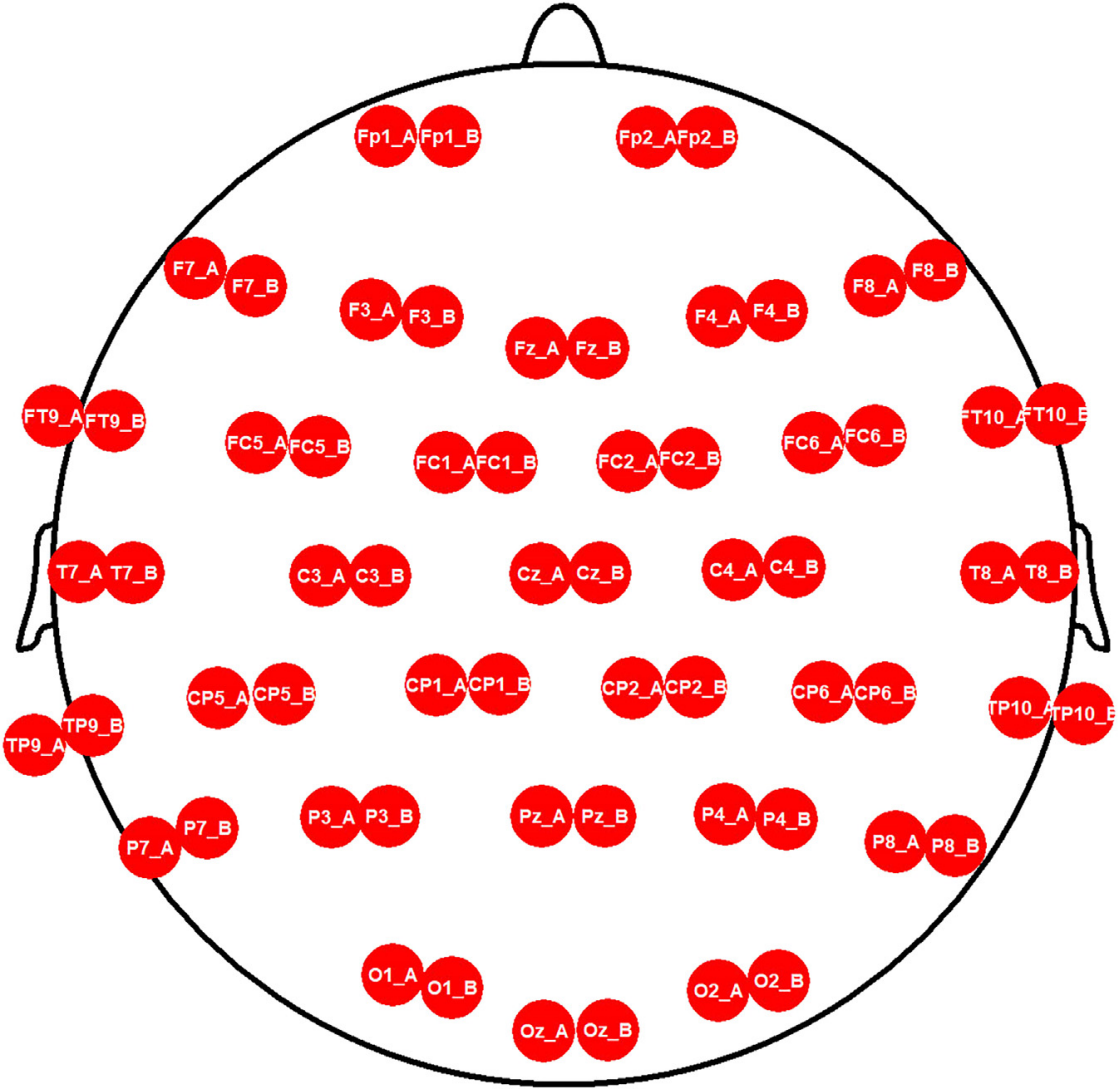
Impedance visualization in BrainVision Recorder for passive electrodes The electrodes of participant A and B are displayed side-by-side.
10. Optional: prepare behavioral questionnaires.
  a. Determine which information could affect your outcome measure (e.g., demographics, participants’ relationship, Empathy Quotient).
  b. Create the questionnaires (e.g., using a survey software such as Qualtrics, or Excel).
  c. Determine if questionnaires will be digital or printed.
11. Prepare participants’ consent forms:
  a. Informed consent with information about the study goal, procedure, and data storage.
  b. Additional consent form for the specific use and dissemination of sensitive data (audio-visual data).

**CRITICAL:** First, consult your institute guidelines and/or templates for participants testing with EEG and for collecting sensitive data.

12. Prepare the instructions for the participants:
  a. Write clear instructions for each part of the experiment.
  b. Decide at which moment of the experimental session the instructions will be given.
  c. Decide how instructions will be given: e.g., the experimenter reads a standard script, on printed paper or digitally on a device.

### Piloting

**Timing: 2–3 weeks**

***Note:*** It is advised to first test both the setup and the experiment materials before starting data collection. Conducting a pilot experiment is a good way to address both points and it gives the possibility to test for other features of the experiment.

13. Define the goals of the pilot experiment.
  ***Note:*** Depending on the goals of the pilot, you may need a different sample size: for instance, to test the equipment, the clarity of the instructions and the duration of the experiment, 2–3 dyads should be enough. However, if you would like to inspect the EEG results, you will need a bigger sample size (at least 10 dyads).
  a. Test the equipment:
    i. EEG acquisition setup.
    ii. Audio-visual equipment (see problem 2 in the troubleshooting section).
    iii. Programmed experiment and EEG recording software (see problem 3 in the troubleshooting section).
  b. Test the instructions:
    i. Evaluate how clear the instructions are, based on the pilot participants’ opinions and task performance.
  c. Test additional experiment features:
    i. Experiment and blocks duration.
    ii. The volume of the stimuli.
    iii. Evaluate if the task elicits the expected conversational features (see problem 4 in the troubleshooting section).
  d. Account for potential confounding factors (see problem 1 in the troubleshooting section):
    i. E.g., Acoustic differences across conditions (face-to-face, back-to-back, face-to-face occluded).
14. Conduct the pilot experiment on a small sample size.
15. Evaluate the pilot results and adapt the experiment accordingly:
  a. If you encounter issues with the setup, check that all the cables are connected correctly, carefully verify that all the steps were followed and check the troubleshooting section.
  b. If the instructions are not clear to the participants, simplify them and make them clearer.
  c. If participants’ task performance is lower than expected, consider decreasing the difficulty of the task.
  d. If the experiment blocks are too long, reduce the amount of trials or the block duration (depending on your experimental design).
  e. If the volume of the stimuli is too high or too low, change the volume level.
  f. If the task is not eliciting the behavior and the results that you were expecting (and this is fundamental for your research question), consider selecting a different task. You could also compare different tasks during the pilot and select the one that you find most suitable for your research goals.

## Key resources table

**Table:** 

REAGENT or RESOURCE	SOURCE	IDENTIFIER
**Deposited data**		

Exemplary data and scripts	This paper	https://osf.io/3dp2e/
Data and code	Drijvers and Holler^9^	https://osf.io/m9rvy/

**Experimental models: Organisms/strains**		

52 Human subjects (32 females; age range = 18–30 years)	Drijvers and Holler^9^	https://www-sciencedirect-com.ru.idm.oclc.org/science/article/pii/S2589004222016856?via%3Dihub
60 Human subjects (45 females; age range = 18–32 years)	Mazzini, Holler, Hagoort, and Drijvers (in preparation)	<identifier will be provided once the article is published>

**Software and algorithms**		

BrainVision Recorder software (version 1.23.0001)	Brain Products	https://www.brainproducts.com/downloads/recorder/
Presentation ® software (version 22.0)	Neurobehavioral Systems	https://www.neurobs.com/menu_presentation/menu_features/features_overview
FieldTrip v20200409	Oostenveld et al.^25^	https://www.fieldtriptoolbox.org/download/
MATLAB v2022b	MathWorks	https://www.mathworks.com/products/matlab.html
Praat version 6.1.42	Boersma and Weenink^18^	https://www.fon.hum.uva.nl/praat/
ELAN version 6.2	Max Planck Institute for Psycholinguistics^33^	https://archive.mpi.nl/tla/elan

**Other**		

Two EEG recording caps (e.g., actiCAP SNAP holders)	Brain Products	https://brainvision.com/products/cap-with-acticap-snap-holders-for-acticap-slim-electrodes/
Two 32 channels electrode sets (e.g., actiCAP slim)	Brain Products	https://www.brainproducts.com/solutions/acticap/
Two control boxes (e.g., actiCAP)	Brain Products	https://www.brainproducts.com/solutions/brainamp/
Two flat ribbon cables	Brain Products	https://shop.brainvision.com/100cm-brainamp-connector-cable/
Two BrainAmp DC amplifiers	Brain Products	https://brainvision.com/products/brainamp-dc/
Two Power Pack rechargeable batteries	Brain Products	https://brainvision.com/products/brainamp-dc/
Two fiber optic cables	Brain Products	https://shop.brainvision.com/fiber-optic-cable-for-brainamp-5m/
One USB2 Adapter, BUA64	Brain Products	https://brainvision.com/products/usb-2-adapter-bua/
One LPT cable	Brain Products	https://shop.brainvision.com/trig26-trigger-cable-lpt-bnc-3m/
One recording computer (HP Z4G4 Xeon 16/512GB) with BrainVision Recorder installed	HP	https://www.hp.com/
One stimulation Computer (HP Z6G4 Xeon Silver 32/512GB) with Presentation ® installed	HP	https://www.hp.com/
Three video cameras (CANON Legria HF G26 or G30)	CANON	https://www.canon.nl
Video cameras SD cards (32 gb, class 10)	Transcend	https://nl.transcend-info.com/
Two microphones (Sennheiser ME-64)	Sennheiser	https://en-us.sennheiser.com/
Two headphones (RP-HT030)	Panasonic	https://www.panasonic.com/nl/
Audio mixer XENYS502	Behringer	https://www.behringer.com/
Audio mixer ALESIS MULTIMIX 4 USB FX	ALESIS	https://www.alesis.com/
Beep generator hardware	Arduino	https://www.arduino.cc/en/hardware/
Electrode conductive gel	EasyCap	https://shop.brainvision.com/v16-super-visc/
Skin preparation scrub (NuPrep)	Weaver and Company	https://www.weaverandcompany.com/products/nuprep/

## Step-by-step method details

### Participants recruitment

**Timing: 1–3 months (for steps 1 to 2)**

This section describes important aspects to consider for participant recruitment.

1. Determine in- and exclusion criteria for participation:
  a. Determine the age range of interest.
  b. Epilepsy should be an exclusion criterion, based on EEG safety guidelines.
  c. Determine language of interest.
    i. Determine the proficiency level of interest: for instance, second-language learners or proficient speakers.
      ***Note:*** For the mentioned studies, only native speakers were selected, since this selection option is available in our recruitment database. Alternatively, LexTALE^21^ is a standardized test of vocabulary knowledge for proficient speakers of English as a second language and it could be used to evaluate participants’ proficiency. However, it is only available for a small set of languages (English, German and Dutch).
  d. Determine potential disorders, which could affect your population results, such as hearing and sight abilities, neurological and language disorders.
  e. Determine if the degree of relationship between conversational partners is an important factor for the research question.
    ***Note:*** Previous research suggested that inter-brain synchrony is influenced by social closeness.^8^^,^^22^ If you would like to investigate inter-brain synchrony under specific conditions, you should first exclude potential effects driven by social closeness.
    i. If it is an important factor: determine the desired degree of relationship (e.g., strangers, acquaintances, friends, family, partners) or treat it as a variable in the analyses by asking participants to fill in a questionnaire.
2. Participant recruitment:
  a. Determine the desired sample size.
    i. The sample size can be calculated with a power analysis or it can be based on previous similar research.
      ***Note:*** For inter-brain analyses, keep in mind that each dyad is treated as a unit of observation and, as a consequence, a larger sample size is needed. Namely, if the common sample size for a standard EEG study is 30 participants, the sample size for a dual-EEG experiment will need to be 30 *dyads* (60 participants) to result in similar power.
  b. Determine if participants will be coupled by the experimenter and/or if they can choose their own experimental partner.
    ***Note:*** If you plan to only include participants with a specific degree of relationship (e.g. friends or partners), the dyad will be already formed and recruited directly. However, if you plan to include also strangers, the experimenter will need to couple them to form a dyad. Consider that recruiting dyads can take longer than recruiting single participants and this could also affect the procedure: opening the experiment to both strangers and acquaintances may speed up the recruitment process. For the experiments on which the current protocol is based, both strangers and acquaintances were included, so they were either matched by the experimenter or one participant would bring along the second participant (e.g. their partner, a friend or a family member).
    i. If participants are coupled by the experimenter: determine if the process will be randomized or determine any relevant criteria for coupling (e.g., age difference, gender).
  c. Develop a specific procedure for participant recruitment:
    i. Prepare standard e-mails to explain the study procedure and risks to interested participants.
    ii. Keep track of the recruitment process, including nr. of tested participants, nr. of interested participants, nr. of excluded participants, and nr. of participants to re-contact.

### Experiment room preparation

**Timing: 45 min (for steps 3 to 7)**

This section describes the preparation of the setup for data collection, including dual-EEG electrode placement.

3. Prepare the experiment room:
  a. Turn on the recording and the stimulation computers.
  b. Turn on the cameras, and check that the SD cards are inside and that the cameras are working well.
  c. Referential communication task:
    i. Switch the microphone on.
    ii. Adjust the volume knob of the audio mixer to the desired level (Figure 5).
  d. Check the condition order, the speaker-listener order, and the speaker-listener position order for the dyad.
  e. Open BrainVision Recorder on the recording computer.
    i. Open the experiment workspace.
  f. Open the program Presentation on the stimulation computer.
    i. Turn off the network connection to improve the precision of the timing of stimuli presentation.
    ii. Open the experiment file and check that all the settings are correct.
4. Prepare the EEG supplies:
  a. Fill conductive gel in two syringes and attach the needles to them.
  b. Prepare scrub (NuPrep), alcohol pads, electrode stickers, cotton pads, white tape (to keep electrodes sticking to the skin), and measuring tape for head circumference.
  c. Insert charged batteries in the two control boxes.
  d. Plug a power pack into each amplifier.
  e. Check and change desired target impedance on the ActiCAP Control software.
    i. Connect one ActiCAP control box (with charged batteries) to the recording computer using the provided USB cable.
    ii. Open the ActiCAP Control software and select Impedance check (Figure 8).

**Figure 8:**
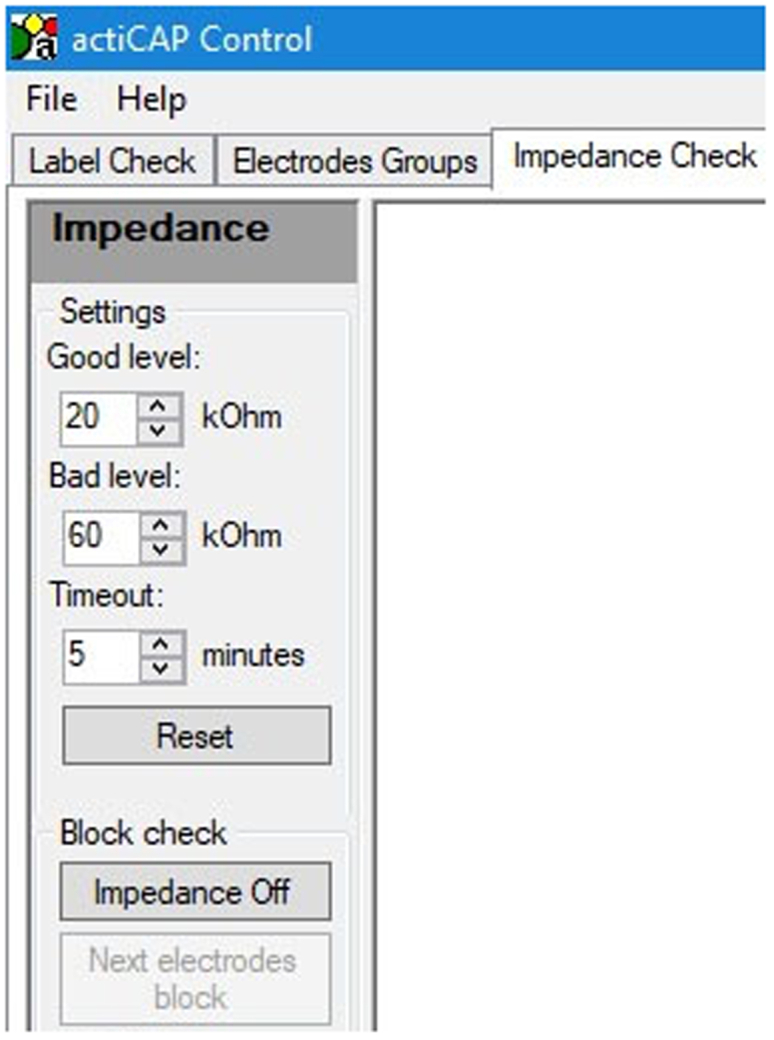
Selection of the target impedances on the ActiCAP Control software
    iii. Click on *Impedance ON.*
    iv. Select the threshold for ‘*Good level’* impedances (e.g., 20 kOhm).
    v. Select after how long the impedance check will time out on the control box (e.g., 5 min).
5. Meet participants:
  a. Ask them to turn off their phones.
  b. Ask them to read the informed consent and give them the opportunity to ask questions.
  c. Give them the opportunity to give their consent to participation and data usage and storage by signing the consent form.
6. Cap and electrode placement. The steps described below need to be performed separately for each participant.
  ***Note:*** Preparing one participant for EEG takes about 25 minutes with 32 electrodes, therefore we suggest having two experimenters available to speed up the process: each experimenter prepares one participant.
  a. Place the reference electrodes: depending on your research question, you may select different reference channels. We used mastoid channels to obtain a symmetrical signal on the scalp and to properly capture activity over the midline.
    i. Use the reference electrode and choose another electrode from the set (e.g., TP10) to be placed on the left and right mastoids. We suggest to re-reference the data to both mastoids during pre-processing, to avoid a hemispheric bias in the recorded activity.
    ii. Clean the skin with scrub and alcohol pads.
    iii. Dry the skin and attach the electrode with a sticker.
    iv. Fill the electrode with conductive gel and distribute it well.
      ***Note:*** An alternative to placing the mastoid electrodes directly on the skin is to insert them in the EEG cap at positions TP9 and TP10. Attaching the mastoid electrode directly to the skin has the advantage of a better and more stable reference signal. The reference position can also be modified depending on your research question.
  b. Measure the participant’s head from nasion (i.e., nasal bridge) to inion (i.e., bony protuberance in the occipital bone) and select the right cap size (e.g., 54 cm/56 cm/58 cm/60 cm, for ActiCAP).
  c. Place the cap on the participant’s head and attach the chin strap: it should not be too tight nor too loose.
  d. Center the cap over the head using the measuring tape: the electrode Cz must be in the middle between nasion and inion and in the middle between left and right ear tops.
  e. Insert the electrodes in the (green) holders of the EEG cap (for ActiCap with 32 electrodes).
  f. For each participant, connect the electrode set cable and the reference and ground cables to the control box.
  g. Reduce impedances:
    i. Start with the reference and the ground electrode: fill the electrode with gel.
    ii. Check the impedances by clicking on the symbol **Z** on the control box (for ActiCAP active electrodes) or by clicking the symbol  on BrainVision Recorder (for passive electrodes).
    iii. In either case, distribute the gel with a circular motion of the needle until the electrode light (active electrodes) or color (passive electrodes) becomes green (below 5kohm for passive electrodes and below 20kohm for active electrodes) (see problem 5 in the troubleshooting section). However, do not insert excessive amount of gel to avoid bridges between electrodes.
    iv. Repeat the same procedure for all the electrodes.
    v. Once the electrode lights are all green, click again on the symbol **Z** on the control box or on the symbol on BrainVision Recorder to interrupt the impedance check.
  h. **Structured dialogue**: place EOG electrodes to record eye movements (Figure 9):
    i. Clean the skin with the scrub and alcohol.
    ii. Dry the skin and place two electrodes above and below one eye (for vertical eye movements) and two electrodes near the outer canthus of each eye (for horizontal eye movements).^13^^,^^23^
    iii. Fill the electrode with gel and distribute it until the desired impedance is reached.

**Figure 9:**
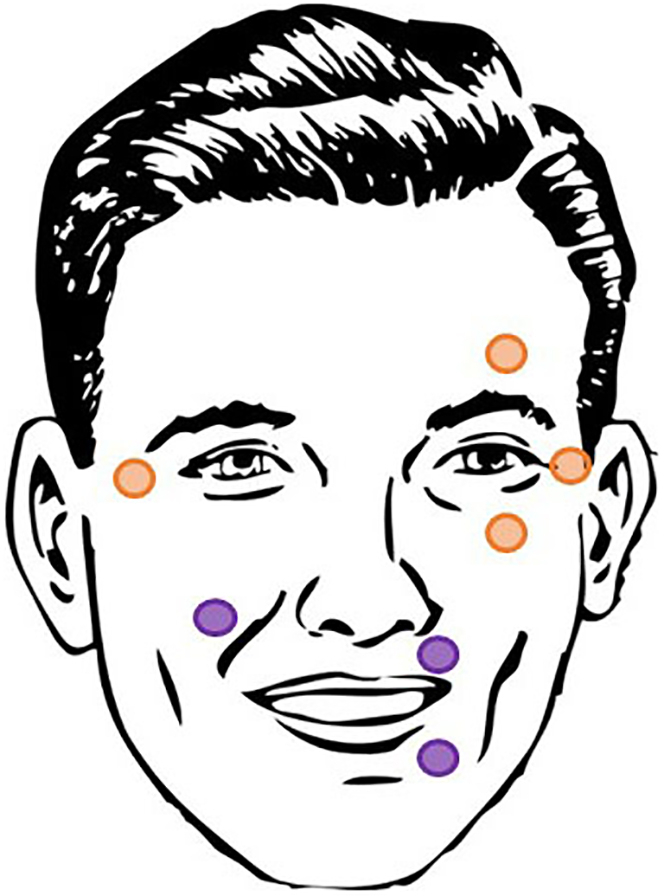
EOG and EMG electrodes placement for the recording of eye (orange) and mouth (purple) movements, respectively
  i. **Referential communication task**: place EMG electrodes to record mouth movements (Figure 9):
    i. Repeat the same procedure for the EMG electrodes.
    ii. Place one electrode near the orbicularis oris superior muscle and one near the orbicularis oris inferior muscle, both on the same side of the mouth.
    iii. Place a third electrode on the right cheek.^24^
7. Prepare the setup to start the recording:
  a. Switch on the two amplifiers and connect the amplifier connection cables to the respective control box.
  b. Ask the participant to stay steady and relaxed, click on the symbol  on BrainVision to display the electrodes’ signal, and check whether the signal is clear and unperturbed.
  c. **Referential communication task**: place the headphones on top of the EEG cap for each participant.
  d. Check the view from each camera: one should point at participant A, one at participant B and one should include both participants.

### Data collection

**Timing: 1 h/dyad (for steps 8 to 15)**

This section describes the experiment procedure to collect dual-EEG and audio-visual data.

8. Give participants instructions:
  a. Explain what they need to do during the experiment (e.g., have a free conversation, plan an event, play a game) and the rules of the task (e.g., what they are and are not allowed to do).
  b. Explain that they should minimize laughing and moving their bodies rhythmically, as to not distort the EEG signal. For participants with previous EEG experience it may be easier to limit their body movements throughout the experiment.
9. Start the experiment:
  a. First, start the video recording on each camera and check that both the video and audio signals are being recorded correctly.
    i. You can check this directly on the camera or on the audio mixer: check if the audio signal changes whenever participants say something.
  b. Start the recording on BrainVision Recorder by clicking on the symbol .
    i. Save the recording with an anonymized acronym name, including dyad code, session and condition (e.g., PP*dyadnumber*_*conditioncode*).
    ii. Check that the EEG is being recorded: on the BrainVision Recorder window, below the EEG signal, you should see the words: .
  c. Inform participants that the experiment is about to start.
  d. Start the experiment on Presentation:
    i. On the stimulation computer, click on the button *‘Run’* on the Main section.
    ii. In the pop-up window click on the button *‘Run scenario’.*
    iii. Insert the block code and press enter.

**CRITICAL:** Check that the triggers are sent correctly to the EEG recording.

10. During the experiment
  a. Check the EEG signal:
    i. If the signal of one or multiple electrodes looks noisy, write it down in the data collection logbook and, if possible, further lower the impedances during experiment breaks.
  b. Check participants’ performance:
    i. If participants are not performing the task correctly, inform them and, if necessary, repeat or clarify the instructions. Remember to pause the experiment before interrupting the participants.
  c. Write down in the data collection logbook any other issues or troubles.
11. At the end of the block
  a. If you specified the length of the block in Presentation, you will see a black screen once the time has run out. If you would like to stop the experiment before the time has run out, click on the space bar on the stimulation computer.
  b. Stop the recordings:
    i. Stop the EEG recording on BrainVision Recorder by clicking on .
    ii. Stop the video recording on each camera.
12. Repeat the same procedure for each block of the experiment.
13. After the experiment
  a. Collect additional data of interest (e.g., behavioral questionnaires)
    i. Ask participants to fill in the questionnaire truthfully and give them specific instructions.
    ii. When the participants finalized the questionnaire, explain to them the goal of the study or give them a debriefing form.
    iii. If you recorded sensitive data and would like to use it in a (scientific) publication and/or at conferences, ask participants to sign a consent form where they inform you how you can use their data. Make sure to check the latest privacy regulations for participants testing in your country on how to handle the data and the participants’ consent.
  b. Remove electrodes and caps:
    i. Remove facial and mastoid electrodes from the participants’ skin and take off the caps with the electrodes.
  c. Restore the experiment room to its initial state:
    i. Switch on the network connection for the stimulation computer.
    ii. Switch off the amplifiers and unplug the cables connecting power packs and amplifiers.
    iii. Charge the power packs.
    iv. Remove the batteries from the control boxes and charge them.
    v. Follow the cleaning procedure of your EEG laboratory.
14. Saving the data:
  a. Check that the EEG recordings were saved correctly.
    i. The EEG recording from BrainVision Recorder consists of three files: the header file (.vhdr), the marker file (.vmrk) and the data file (.eeg). Check that the marker file contains the triggers information in the desired order.
  b. Save the questionnaire data with an anonymized acronym.
  c. Remove the SD cards from the camera and save the videos using an anonymized acronym.
    i. Check that the video and audio have been recorded correctly (e.g., you can see and hear both participants well).
    ii. Once the videos have been backed up, erase the video recordings from the SD cards and insert the cards back into the cameras.

***Note:*** Check your institute’s guidelines regarding the anonymization of the files. In some cases, it is acceptable to use the same acronym for different files, in other cases it is not acceptable.

15. Audio-visual data pre-processing:
  a. If you plan to analyze the EEG data in relation to the audio-visual data, make sure to trim the audio and the video data so that they start at the EEG recording start cue.

## Expected outcomes

The protocol outcome consists of a dataset including synchronized dual-EEG data and audio-visual data. The dual-EEG recordings will show electrophysiological activity of two interacting participants at the same time (32 channels per participant) with marked relevant events. The audio-visual data will include two/three (e.g., structured dialogue/referential communication task) separate video recordings (capturing different camera views) including participants' visual (e.g., gestures, movement, facial expression) and auditory behavior (e.g., speech). Additionally, participants demographics and Emotional Quotient scores are collected by means of behavioral questionnaires.

The collected data will be suitable for several types of analyses, such as conversation and gesture analyses, intra-brain spectral and time-frequency analyses, as well as intra- and inter-brain connectivity and coherence analyses (e.g., cortical tracking of speech and inter-brain synchrony). Depending on the analysis of choice, different results can be expected.

For both tasks described in the protocol, the collected behavioral data should include a rich, naturalistic and multimodal conversation corpus, including disfluencies, facial expressions and gestures. For the referential communication task, a higher occurrence of iconic gestures can be expected, which participants tend to use to describe the characteristics of the referents (i.e., figures).

The structured dialogue paradigm by Drijvers and Holler^9^ used to investigate the influence of spatial orientation and the visibility of body signals on intra- and inter-brain neural synchrony included three analyses steps: intra-brain spectral analysis in delta and alpha frequency bands, intra-brain coherence analysis in delta frequency band and inter-brain spectral and coherence analyses in beta frequency band. Following the paradigm and the related analyses steps, the expected results include a higher engagement (i.e., alpha power) in the face-to-face conditions, a higher cortical tracking of speech for back-to-back conditions compared to face-to-face conditions and a further increase in the condition in which bodily signals are visible. A similar pattern is expected for inter-brain synchrony in the beta frequency.

## Quantification and statistical analysis

Considering that dual-EEG is a relatively recent technique and that there is not yet a standard approach to analyze inter-brain dynamics, we provide an overview of the main data pre-processing and analysis steps using FieldTrip toolbox (v20200409)^25^ running in Matlab (version 2022b).^26^ For a more detailed pre-processing and analysis pipeline using Python,^27^ consult Ayrolles et al.^28^ By recording the neural activity of multiple participants at the same time, dual-EEG opens the possibility to study neural dynamics of interacting brains and this is commonly achieved by means of inter-brain coherence analyses.

1. Pre-processing (Figure 10, see key resources table for example scripts):
  a. Start FieldTrip and load the EEG raw dataset.
  b. Segment the raw continuous data into epochs: choose the event markers you are interested in and the length of the epochs.
  c. Pre-process the data with *ft_preprocessing*: here you can specify filters and re-reference settings. With this function, you can also separate the data of each participants by selecting the channels of participant A and participant B, respectively (see Figure 10). This step is particularly relevant in preparation for artifact rejection and blind source separation, for which it is necessary to analyze the data of each participant separately.
  d. Inspect the data of each participant for artifacts and reject the trials and/or channels affected by major artifacts.
  e. Compute the independent component analysis and select the desired method. If you would like to specifically identify (and exclude) muscle and speech-related components, we suggest the implementation of Blind Source Separation - Canonical Correlation Analysis.^29^^,^^30^
  f. Select and reject components which represent clear noise sources (e.g., eye movements, eye blinks, muscle and cardiac artifacts) and save the pre-processed dataset for participant A and participant B.

**Figure 10:**
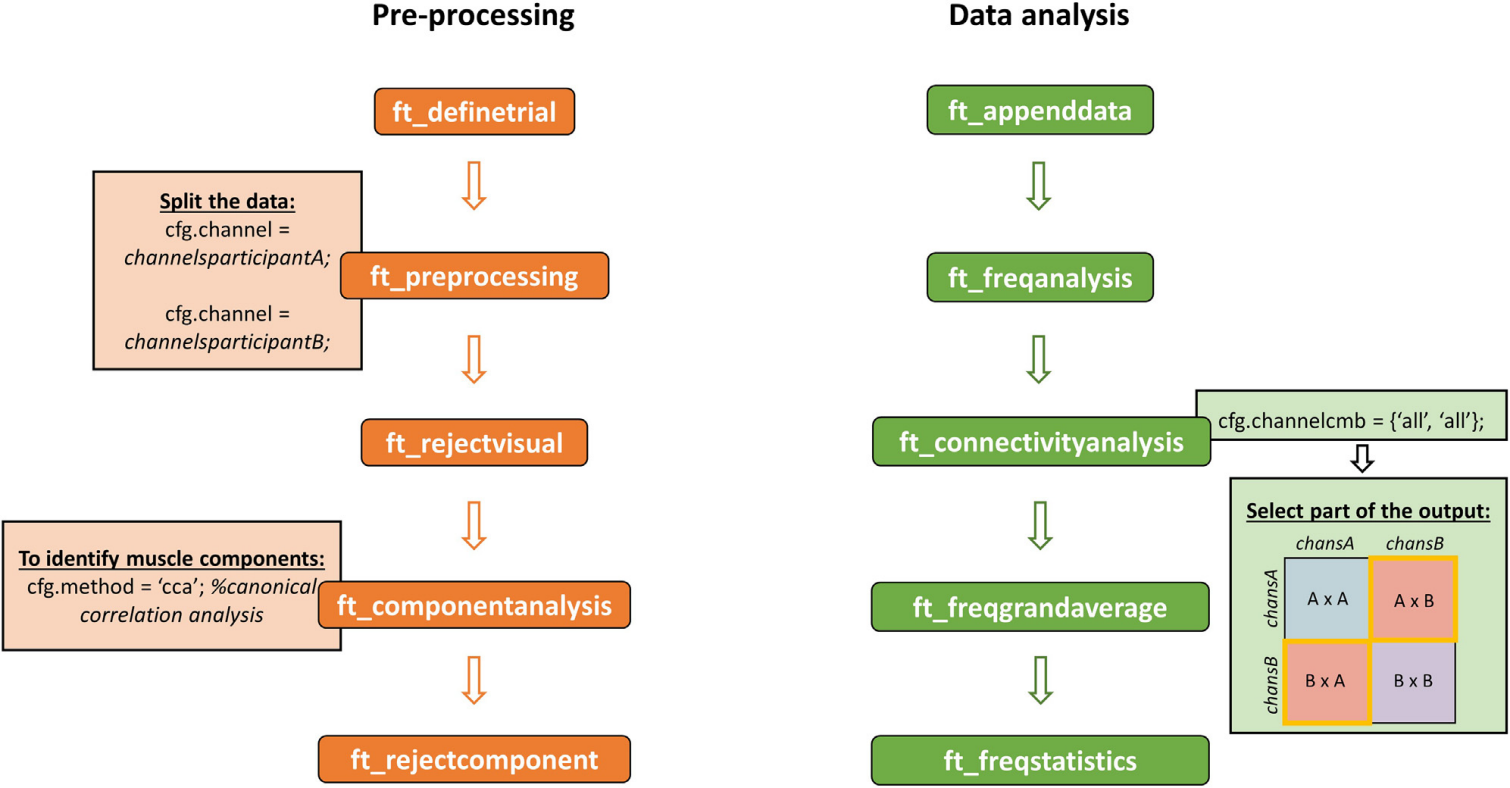
Overview of pre-processing and (inter-brain) data analysis steps for dual-EEG data Drijvers & Holler^9^ provides the data and scripts generated during the study. Example scripts and data specifically related to this paper are also provided (see key resources table).
2. Inter-brain coherence analysis
  a. Merge the pre-processed data of participant A and participant B using the function *ft_appenddata.* Make sure to only include trials that are common to both participant A and participant B: in this way the data will be appended on the channel dimension, resulting in 64 channels (the first 32 for participant A and the following 32 for participant B).
  b. Extract the spectral representations for each condition per participant with *ft_freqanalysis.*
  c. Use the spectral representations to conduct a coherence analysis per participant with *ft_connectivityanalysis.*
    i. Select the desired coherence measures. For an overview of the possible coherence measures, refer to Bastos & Schoffelen^31^ and Ayrolles et al.^28^
    ii. Specify that all channels should be compared with each other. As a result, you will obtain a 64x64 coherence matrix, including both intra-brain and inter-brain coherence values (see Figure 10).
    iii. Select the quarter of the matrix which includes inter-brain coherence values (e.g., in Figure 10: upper left quarter or lower right quarter) and save it.
  d. From the selected quarter of the coherence matrix of each participant, compute the grand average coherence values per condition.
  e. Evaluate the differences across condition with cluster-based permutation tests using the function *ft_freqstatistics.*

## Limitations

The current protocol was developed to study human communication using a more naturalistic approach and, as a result, with less experimental control compared to standard laboratory studies. The idea behind this choice was to elicit and study spontaneous interaction and behavior. For this reason, the protocol might not be suitable for experiments that would require larger experimental control or more complicated tasks and/or stimuli.

By reducing experimental control over participants’ behavior, the EEG data may look noisier in comparison to very controlled experiments. However, this limitation can be minimized both by instructing participants to limit their movements and with specific pre-processing techniques, aiming at excluding muscle-related noisy activity such as Blind Source Separation - Canonical Correlation Analysis.^29^^,^^30^

Lastly, for inter-brain analyses, each dyad represents a unit of observation and, as a consequence, a larger sample size is needed in comparison to standard EEG studies. Considering this and the challenge of recruiting two participants per session, the recruitment process may be difficult and longer than expected. We therefore suggest planning some extra time for participants recruitment.

## Troubleshooting

### Problem 1

Measuring inter-brain neural synchrony or inter-subject correlation? (related to *Experimental design section, step 6b*)

When investigating inter-brain synchrony, there is an important confound to keep in mind: the similarity in brain activity between participants may also be elicited by similar external stimulation.^17^ More specifically, when two participants are presented with the same stimuli (e.g., movie, pictures or sounds), their activity may look similar due to similar underlying cognitive processes. However, if the focus of the investigation is inter-brain synchrony, specifically elicited by joint action and/or social interaction, such potential confounding factors should be excluded.

### Potential solution

**Figure 11:**
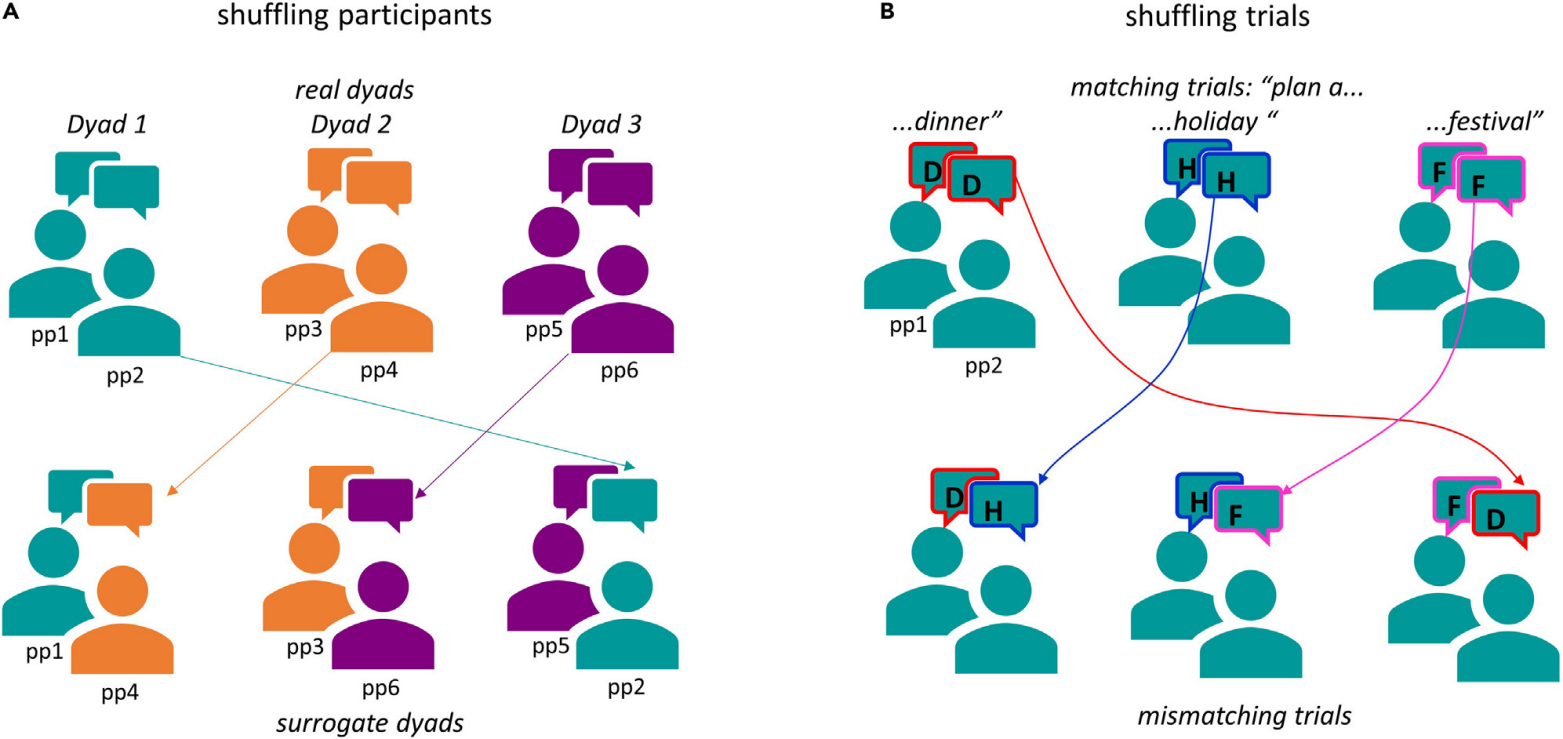
Creating surrogate data to compare to actual data in order to exclude potential confounds (A) Create surrogate dyads by shuffling participants dyads. (B) Create surrogate trials by shuffling the trial order, in other words comparing mismatching trials across participants.

• Avoid comparing conditions involving the presence and absence of external stimulation (e.g., movie, pictures). If inter-brain synchrony is higher in the external stimulation condition, it will not be possible to disentangle between inter-brain activity elicited by external stimulation and by mutual engagement.
• One possibility to circumvent this issue is to select conditions with similar stimuli and manipulate only the independent variable (e.g., spatial orientation, as in Drijvers & Holler,^9^ or relationship type, as in Kinreich et al.^22^).
• Another possibility to exclude the confound of inter-subject correlation is by comparing real data with surrogate data (Figure 11). The surrogate data can be created in two ways: 1) by shuffling the trials, comparing the brain activity of the participants across mismatching trials^3^^,^^26^ and 2) by shuffling the participants forming the dyad,^32^ in this way participants who did not interact together will be compared. Following one of these procedures, if inter-brain synchrony is elicited by external stimuli or by the environment, it should not differ for mismatching trials or for participants, who attended to the same stimuli in the same environment, but did not interact together (at all, or comparing different phases of the interaction). On the other hand, if inter-brain synchrony is elicited by mutual engagement and/or social interaction, this should be trial- and dyad-specific.

### Problem 2

The video recordings do not contain any audio signal (related to *Piloting section, Step 13aii*).

### Potential solution

• Make sure the external microphones are selected as input signals on the camera settings.
• Make sure the audio mixers are turned on and all the cables are well connected.
• Check that the audio signal is modulated both on the audio mixers and on the cameras whenever someone is speaking.

### Problem 3

Event triggers are not sent to BrainVision Recorder (related to *Piloting section, Step 13aiii*).

### Potential solution

• Make sure the recording computer is listed as an output port on the Presentation software port settings. You can test the port after editing the settings.
• Sending multiple event triggers in close succession (i.e., only a few milliseconds in between) can cause errors, such as missing event triggers. In case it is necessary to send multiple triggers at the same time, make sure to include a delay in between them (e.g., 20 ms): wait interval(20);

### Problem 4

Participants do not respect the structured turn-taking, based on the auditory cues *(related to Piloting section, Step 13c*).

### Potential solution

**Figure 12:**
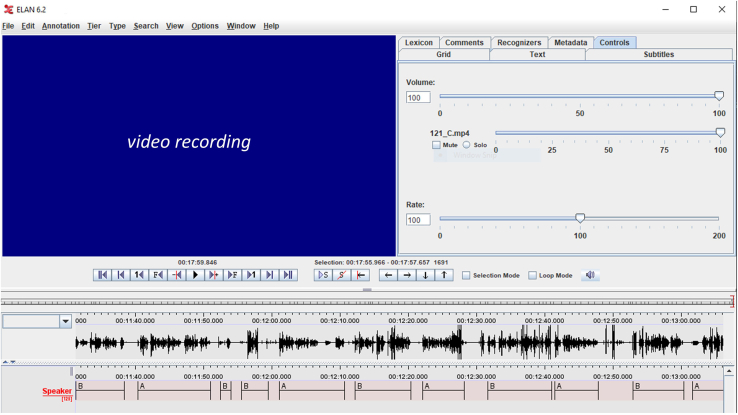
Example of ELAN annotation tool The tool is used to annotate the speaker turns with respect to the speech recording (speech envelope is illustrated) and to the video recording (here not shown due to privacy regulations).

• Remind the participants to attend to the auditory cues. You can pause the experiment in Presentation by pressing **‘P’** on the keyboard and resume it by pressing **‘R’** (check your Presentation general settings beforehand).
• You can check that the instructions are clear and that the task is not difficult enough when piloting the experiment. However, consider that there may be subjective differences in how well participants respect the turn-taking.
• If respecting a specific turn-taking structure is particularly relevant for your research question, but participants fail to follow it, their turns can be annotated offline from the audio(-video) recording with an annotation tool, such as ELAN^33^ or Praat^18^ and thus fed into analyses (Figure 12).

### Problem 5

Certain electrodes do not reach a good impedance level (e.g., > 25 kOhm) after gel application, (related to *Step 6g*).

### Potential solution

• First, make sure that the reference and the ground electrodes have good impedances.
• Repeat the gel application procedure: spread the gel in a circular motion, as you retract the needle, inject more gel, and let it sit for a few minutes.
• Keep in mind that electrodes on the back of the head might require more time to reach good impedances, because of the amount of hair. If you wish to avoid this issue, you may consider using a chest belt set for EEG caps, to adjust for different head shapes.
• If the impedances do not improve for a certain electrode and neighboring electrodes have good impedances, the electrode might be broken. Follow the manufacturer’s guidelines to check and replace the electrode if necessary.

## Resource availability

### Lead contact

Further information and requests for resources and reagents should be directed to and will be fulfilled by the lead contact, Linda Drijvers (Linda.Drijvers@mpi.nl).

### Materials availability

This study did not generate new unique reagents.

## Data Availability

The published article by Drijvers & Holler,^9^ on which the current protocol was mainly based, includes data and code generated during the study. Additionally, exemplary data and scripts are available via https://osf.io/3dp2e/. Any additional information required to implement this protocol is available from the lead contact upon request.
