# Supplementary material for: Studying naturalistic human communication using dual-EEG and audio-visual recordings
Source: STAR Protoc. 2023 Jul 7;4(3):102370. doi: 10.1016/j.xpro.2023.102370 (PMC10511849; doi:10.1016/j.xpro.2023.102370)
Supplement: Document S1. Figures S1–S3 [file mmc1.pdf]

Supplemental materials

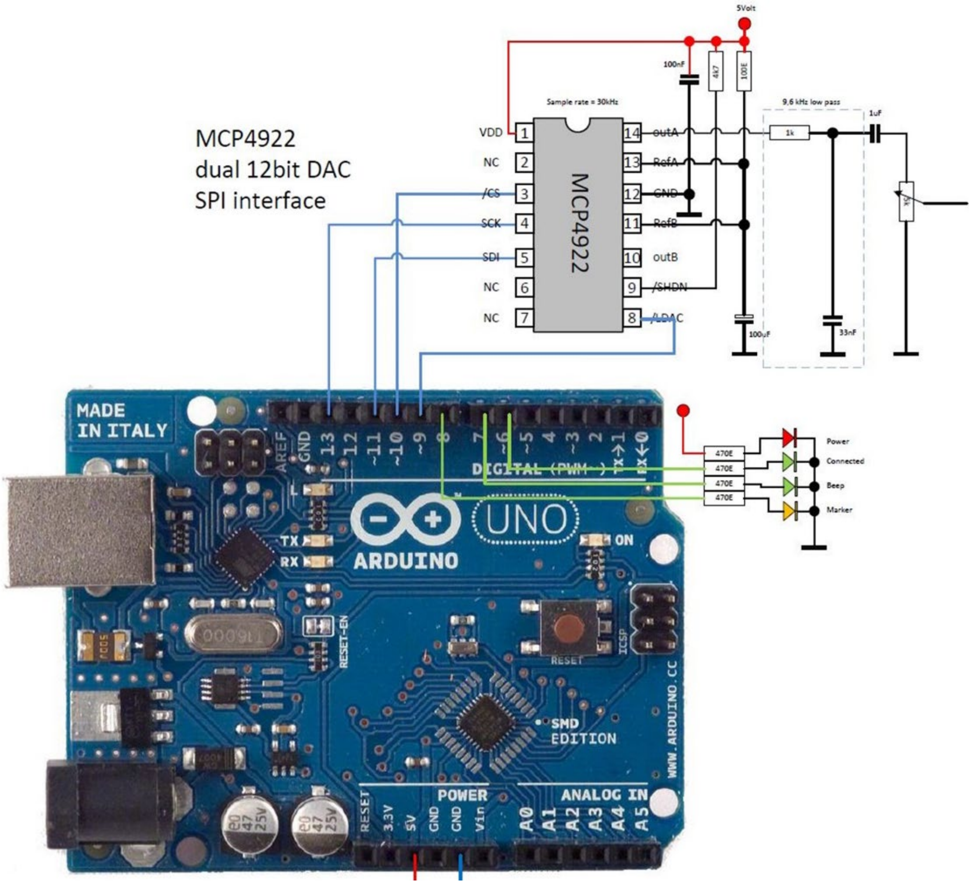

Figure S1: Schematic figure illustrating how to build an automated beep generator device using Arduino UNO hardware; related to Set-up preparation section, step 5.

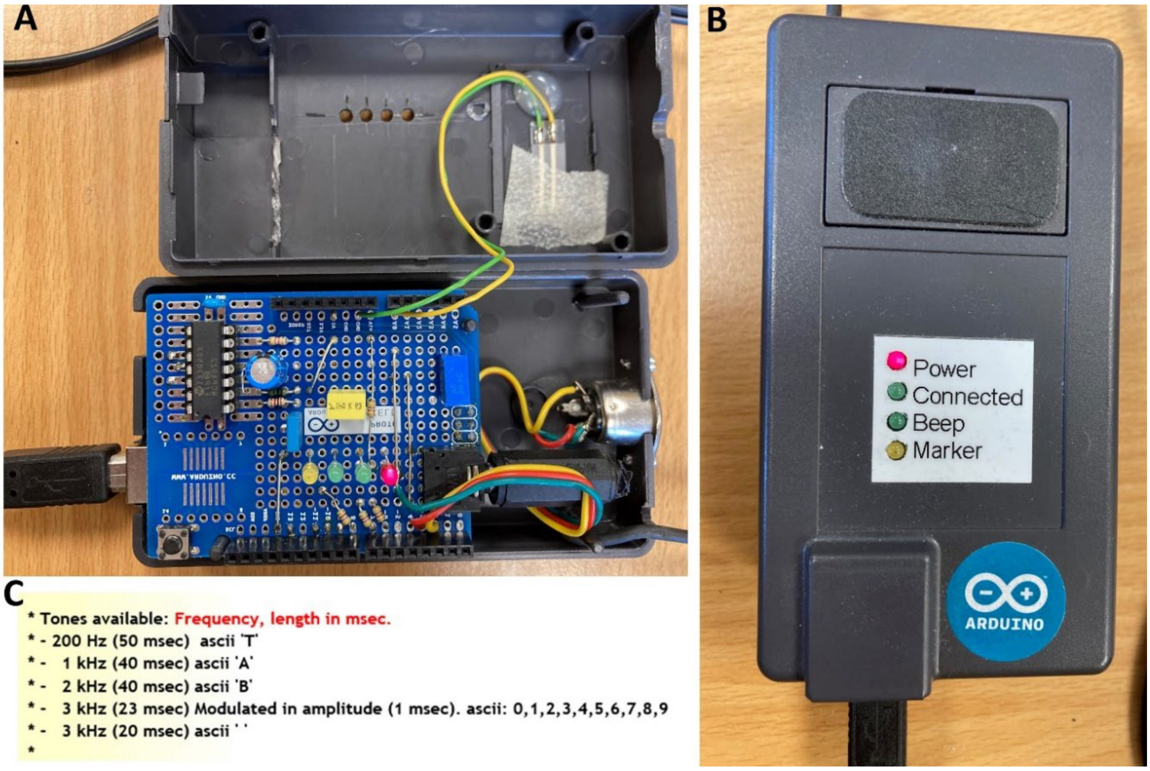

Figure S2: Automated beep generator final result: A: inside view, B: outside view, C: available tones. *An example .wav file with the available tones is provided (see key resources table); related to Set-up preparation section, step 5.*

	Condition			Conversation topic: plan a...			Who speaks first?		
**Dyad**	**Block 1**	**Block 2**	**Block 3**	**Block 1**	**Block 2**	**Block 3**	**Block 1**	**Block 2**	**Block 3**
1	FtF	FtFO	BtB	dinner	festival	holiday	A	B	A
2	FtFO	BtB	FtF	festival	holiday	dinner	B	A	B
3	BtB	FtF	FtFO	holiday	dinner	festival	A	B	A
4	FtF	FtFO	BtB	dinner	festival	holiday	B	A	B
5	FtFO	BtB	FtF	festival	holiday	dinner	A	B	A
6	BtB	FtF	FtFO	holiday	dinner	festival	B	A	B
7	FtF	FtFO	BtB	dinner	festival	holiday	A	B	A
8	FtFO	BtB	FtF	festival	holiday	dinner	B	A	B
9	BtB	FtF	FtFO	holiday	dinner	festival	A	B	A
10	FtF	FtFO	BtB	dinner	festival	holiday	B	A	B
11	FtFO	BtB	FtF	festival	holiday	dinner	A	B	A
12	BtB	FtF	FtFO	holiday	dinner	festival	B	A	B
13	FtF	FtFO	BtB	dinner	festival	holiday	A	B	A
14	FtFO	BtB	FtF	festival	holiday	dinner	B	A	B
15	BtB	FtF	FtFO	holiday	dinner	festival	A	B	A
16	FtF	FtFO	BtB	dinner	festival	holiday	B	A	B
17	FtFO	BtB	FtF	festival	holiday	dinner	A	B	A
18	BtB	FtF	FtFO	holiday	dinner	festival	B	A	B
19	FtF	FtFO	BtB	dinner	festival	holiday	A	B	A
20	FtFO	BtB	FtF	festival	holiday	dinner	B	A	B
21	BtB	FtF	FtFO	holiday	dinner	festival	A	B	A
22	FtF	FtFO	BtB	dinner	festival	holiday	B	A	B
23	FtFO	BtB	FtF	festival	holiday	dinner	A	B	A
24	BtB	FtF	FtFO	holiday	dinner	festival	B	A	B
25	FtF	FtFO	BtB	dinner	festival	holiday	A	B	A
26	FtFO	BtB	FtF	festival	holiday	dinner	B	A	B
27	BtB	FtF	FtFO	holiday	dinner	festival	A	B	A
28	FtF	FtFO	BtB	dinner	festival	holiday	B	A	B
29	FtFO	BtB	FtF	festival	holiday	dinner	A	B	A
30	BtB	FtF	FtFO	holiday	dinner	festival	B	A	B

Figure S3: Randomization for the structured dialogue experiment. *The randomization was done across dyads on Microsoft Excel for the visibility conditions (face-to-face, back-to-back and face-to-face occluded), for the topic of conversation (planning a dinner, a festival or a holiday) and for the first speaker (participant A or B); related to Experiment design section, step 6e.*
